# Spontaneous Rupture of Mature Teratoma at the Cerebellar Vermis Comorbid With Dermal Sinus Tract and Subcutaneous Lipoma

**DOI:** 10.7759/cureus.67634

**Published:** 2024-08-23

**Authors:** Zhiyu Xi, Songsong Lu

**Affiliations:** 1 Neurological Surgery, The First Affiliated Hospital of University of Science and Technology of China (USTC), Anhui, CHN; 2 Neurological Surgery, The First Affiliated Hospital of University of Science and Technology of China (USTC), Hefei, CHN

**Keywords:** case report, rupture, lipoma, dermal sinus tract, intracranial teratomat

## Abstract

Intracranial teratoma, a subtype of non-germinomatous germ cell tumors, is rare in adults. Clinical presentation of intracranial teratomas varies according to where they grow. In particular, cases of spontaneous ruptures of intracranial teratoma are sporadic. This study reports the case of an adult with a spontaneously ruptured mature teratoma in the cerebellar vermis, which was comorbid with a dermal sinus tract and subcutaneous lipoma. Before surgery, because the images were atypical of a teratoma, the patient was misdiagnosed as having vascular malformation rupture and bleeding in the cerebellar vermis. Due to the patient’s level of consciousness dropping drastically to a coma, a craniotomy was performed. During the surgery, the tumor was observed to be a mixed cystic and solid mass. The liquid in the cyst was dark green and with a fatty component. The solid part had a tough texture and comprised hair, fat, cartilage, and calcification components. Post-surgery multipoint biopsy proved that it was a mature teratoma and that it was connected to a subcutaneous lipoma through the dermal sinus tract across the occipital bone. After proactive treatment, the patient’s prognosis was favorable.

## Introduction

Intracranial teratomas originated from primordial germ cells, are a relatively rare subtype of non-germinomatous germ cell tumors and are more common in infants and young children. Accounting for 0.3%-0.6% of intracranial tumors, they often grow in central line regions, such as the pineal region, suprasellar region, and third ventricle [1,2]. Teratomas are classified as mature, immature, and malignant teratomas depending on the degree of differentiation [3-5]. Precise diagnosis and classification are critical to the choice of treatment for the patient and prognosis evaluation. Dermal sinus tracts often occur in the lumbosacral region and coexist with a tethered spinal cord. Because of the existence of the sinus tract and ostium, skin bacteria can colonize the subdural space. This may result in meningitis or encephalopyosis, which are difficult to cure [6-10].

## Case presentation

History and examination

**Figure 1:**
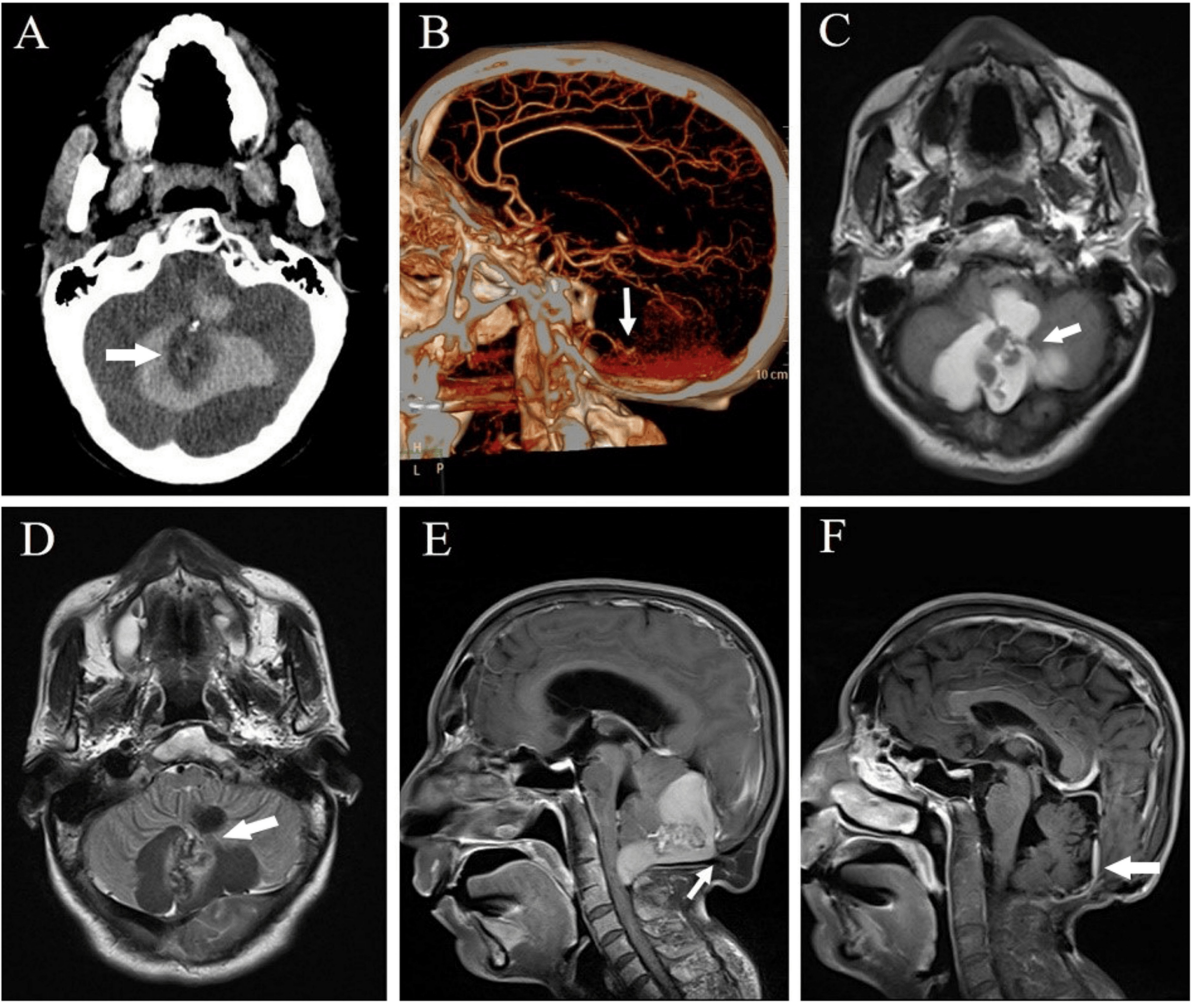
Images of the patient (A) CT scan showing that the lesion in the cerebellar vermis was leaf-shaped. The center exhibited a mixed density of high and low signals, whereas the vicinity was even, high-density signals. (B) CTA image depicting that the ends of the posterior inferior cerebellar arteries were disarranged. A suspicious vascular malformation was found locally (signaled by the arrow). (C) T1WI MRI illustrating that the lesion was leaf-shaped. The center had a mixed density of high and low signals, whereas the vicinity demonstrated even, high-density signals. (D) T2WI MRI illustrating the lesion in the cerebellar vermis. The center exhibited a mixed density of high and low signals, whereas the vicinity was even, low-density signals. (E) T1 enhanced MRI sagittal image depicting that the tumor was situated behind the cerebellar vermis. The center of the lesion exhibited strengthened in a strip shape. The high signal substance in the vicinity was pressed forward by the cisterna magna against the brain stem and the arachnoid foramen of the fourth ventricle. (F) Post-surgery T1-enhanced MRI indicating that the tumor had been completely removed.

A 41-year-old man was hospitalized because he had been experiencing a headache for two days and abruptly fell into a coma for four hours previously. He had a history of meningitis and congenital scoliosis. Physical examination revealed he had a coma and meningeal irritation was positive. Brain computed tomography (CT) revealed a leaf-shaped lesion in part of the cerebellar vermis; the center was a mixed-density signal, whereas the vicinity was a high-density signal (Figure 1A). Furthermore, CT angiography revealed the possibility of vascular malformation at the terminal of the posterior inferior cerebellar arteries (Figure 1B). Brain magnetic resonance imaging (MRI) revealed the lobulation of the lesion in the posterior fossa. The center of the lesion showed a heterogeneous hypointense signal on T1WI and a mixed hyperintense and hypointense signal on T2WI. In contrast, the vicinity had a hyperintense signal on T1WI and a hypointense signal on T2WI (Figures 1C, 1D). Gadolinium-enhanced MRI revealed the center of the lesion exhibited strengthened in a strip shape (Figure 1E). The primary diagnosis based on the patient's images was arteriovenous malformation and bleeding in the cerebellar vermis. The tumor stroke was waiting to be considered.

Operation

**Figure 2:**
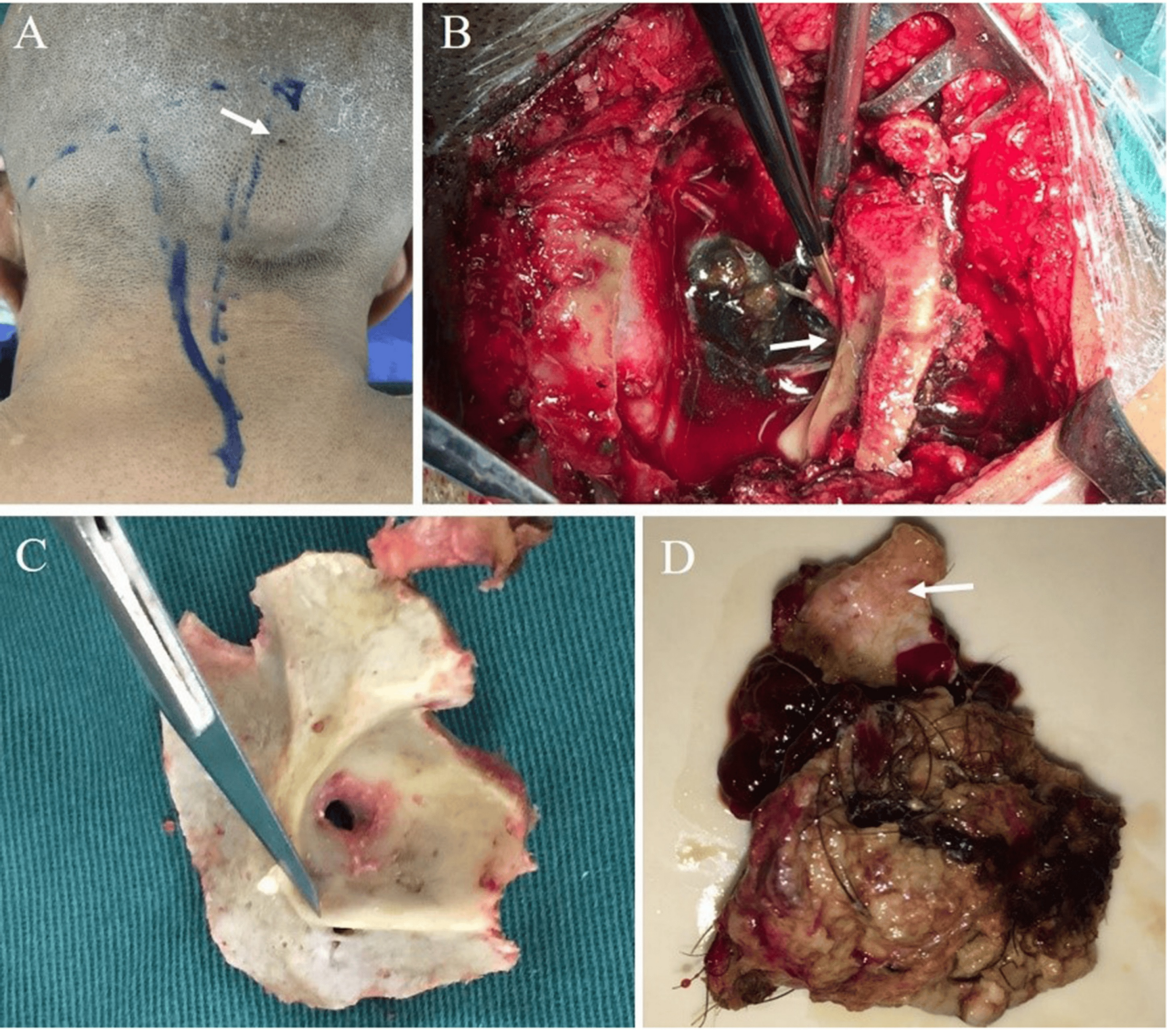
Intra-operative images (A) The patient was lying face down. The surgery used the suboccipital posterior midline approach. A subcutaneous swell at the occiput was observed, on the surface of which a 2-mm ostium was situated (indicated by the arrow). (B) During the surgery, the bone flap and dura mater were opened. The tumor was surrounded by an incomplete capsule, had a mixed cystic and solid mass, and contained dark-green cystic liquid. The local dura mater passed the sinus in the occipital bone and was tightly adhered to the subcutaneous tissue (indicated by the arrow). (C) When the occipital bone flap was removed, the dermal sinus tract was observed. (D) The cystic part of the tumor was completely removed; its surface comprised hair, fat tissue, and cartilage tissue components (as indicated by the arrow).

Resection was performed with a suboccipital posterior midline approach. When designing the incision, we observed a subcutaneous swell at the occiput. Its surface had a dot-shaped ostium with a diameter of approximately 2 mm (Figure 2A). After we cut open the occipital skin, a lipoma was found. Its capsule was connected to the inside of the skull through the occipital sinus tract. After removing the bone flap, the dura mater was tightly stuck with the lipoma capsule through the sinus tract. After the dura mater was opened, a considerable amount of dark-green oily substance flowed out (Figure 2B). After carefully removing the liquid secretion, the solid part of the tumor was observed. It had an oval shape, was surrounded by an incomplete capsule, and was tightly connected to the surrounding arachnoid mater. The tumor boundary was clear. No obvious blood supply was found. The tumor compressed the cerebellar vermis; its base was closely connected to the posterior inferior cerebellar arteries and their branches. We carefully dissected the arachnoid mater and blood vessels adhered to the tumor. The tumor was removed along with its connected arachnoid mater. In the removed tumor, components of fat, hair, cartilage, and calcification were observed (Figure 2D). The dura connected to the sinus tract was cut off (Figure 2C), and the dural defect was repaired by fascial tissues. Furthermore, the inner wall tissue in the sinus tract in the occipital bone was cleaned. The occipital bone was cleaned by soaking it in iodine and reset.

Histological analysis and postoperative course

**Figure 3:**
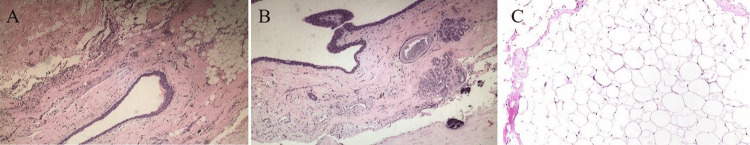
Histological images Pathological findings of the mature teratoma and subcutaneous lipoma. Low-magnification (x100; A and B) and high-magnification (x400; C) photomicrographs of H&E-stained sections. (A, B) Testing from the immunohistochemistry revealed that the main component of the lesion was the squamous epithelium, originating from the ectoderm. The inside of the lesion contained glands from the mesoderm. (C) A large number of adipocytes can be seen from the pathological section of the subcutaneous lesion, which indicates leiomyoma.

The results of immunohistochemistry revealed the tumor in the cerebellar vermis was a mature teratoma (Figures 3A, 3B). Furthermore, the subcutaneous tumor was a differentiated, mature lipoma (Figure 3C). The patient's consciousness was restored, and none of his neurofunctions were impaired. Brain MRI showed the tumor had been completely removed (Figure 1F). We retrospectively reviewed the brain CT scan and found that the center of the lesion exhibited a small high-density signal, which was a bone structure. The leaf-shaped high-density signal in the vicinity was revealed to be lipid secretions (Figure 1A). Brain MRI revealed the mixed signals at the center of the lesion were a solid tumor structure, and its vicinity was the cystic part of the tumor. A sagittal image revealed an occipital subcutaneous lipoma, which was connected to the teratoma through a tube-shaped structure, namely, the dermal sinus tract (Figure 1E). We followed up on the patient six months after surgery and observed no sign of tumor recurrence.

## Discussion

This case was rare because of the comorbidity with the dermal sinus tract and subcutaneous lipoma; to date, no such case has been reported. The formation mechanism of a mature teratoma comorbid with a dermal sinus tract remains unclear. The skin and nervous tissues originate from the ectoderm. During the fetal period, if the fetus's nervous ectoderm and epidermal ectoderm are not completely separated, a dermal sinus tract may occur on the dorsal side of the cerebral-spinal axis, specifically, anywhere from the occipital bone to the lumbosacral vertebra [7,8]. Generally, the inner side of the dermal sinus tract is connected to a dermoid or epithelioid cyst. In this case, however, preoperative brain CT and MRI revealed the solid part of the tumor contained a calcified component, whereas the cystic part comprised a liquid fatty substance. During surgery, hair and cartilage tissues were found within the tumor. Postoperative biopsy proved that it was a mature teratoma. Existing theories regarding the occurrence mechanisms of intracranial teratomas include the abnormal distribution of primordial germ cells as well as gene mutation [11,12]. In the past, rupture cases of dermoid cysts, epidermoid cysts, and teratoma in the central nervous system were rare [13,14]. The reason behind the rupture remains unclear. Stendel [15] proposed a hormone theory and stated that the glandular secretion function of the cyst wall may increase because of the change in hormone secretion in patients with age growth, resulting in the rapid enlargement and rupture of cysts. However, the patient in our case was middle-aged, during which period his hormone levels should be relatively stable. Therefore, the hormone theory is insufficient to explain this phenomenon alone. Lunardi and Missori [16] asserted that the spontaneous rupture of cysts was related to head movements, particularly the pulsation of brain tissues. The water hammer effect may cause creaks on the cyst wall and the overflow of its contents. We contend that cyst walls have uneven thicknesses and different surrounding supportive structures. As the content of the cyst increases, local pressure at the weak points of the cyst wall is increased. In addition, the combined mechanical stress of brain pulses, head movements, and head injuries may cause the cyst wall to rupture.

Clinical presentations of the rupture of a mature teratoma or dermoid cyst are typically related to cystic fluid leakage. The leaked lipid may cause aseptic inflammation. The stimulus from lipid substances may trigger epilepsy, vasospasm, or cerebral hemorrhage. Furthermore, granulomatous inflammatory response at the outlet of the fourth ventricle may trigger hydrocephalus [17,18]. In our case, MRI revealed the tumor had spontaneously ruptured, and lipid substances had leaked into the cisterna magna, pressing the fourth ventricle and brain stem, thereby affecting the patient's level of consciousness. Thus, the disease progression was fast and similar to that of acute hemorrhagic stroke. During surgery, once the dura mater had been opened, a dark green oily substance poured out. Moreover, the tumor was surrounded by an incomplete capsule. Most scholars have asserted that the fat and calcification components in a tumor are the keys to diagnosing a teratoma. If a teratoma ruptures, the lipid substance or drips of cholesterol can enter the subarachnoid cavity or the ventricular system. Thus, there was a fat-liquid plane observed in the subarachnoid cavity or ventricular system while the patients' posture changed [19]. In our patient, the teratoma occurred at the back of the cerebellar vermis, the other side was restricted by the cisterna magna arachnoid mater. After the rupture, the lipid only compressed local structures and did not enter the subarachnoid cavity. Consequently, the typical fat-liquid plane was not observed. The preoperative images of this patient were atypical, and the CT scan revealed high-density signals in the posterior cranial fossa, and CT angiography revealed the lesion was closely related to the posterior inferior cerebellar arteries, the patient was misdiagnosed as small vascular malformation. Post-surgery retrospective image analysis indicated the unique characteristics of a mature teratoma. First, CT revealed the lesion exhibited leaf-shaped changes, which differed from the oval mass effect by hematomas. Next, an MRI revealed the lesion was outside the brain parenchyma, which was different from hematomas in brain parenchyma caused by the rupture of vascular malformation. The axial and sagittal planes of the MRI showed the capsule of the tumor passed the occipital bone through the strip-shaped dermal sinus tract and connected to the subcutaneous lipoma. Therefore, integrating and analyzing CT and MRI images to search for characteristics can increase the accuracy of preoperative diagnoses.

The precise diagnosis and classification of a teratoma are critical for selecting treatment methods and evaluating the prognosis [1,4]. Mature teratoma can be completely removed through surgery to cure the patient. By comparison, immature and malignant teratomas that have undifferentiated embryonic tissues, primitive germ cells, or choriocarcinoma components may reoccur even if they are completely removed. Therefore, postoperative radiotherapy combined with chemotherapy should be conducted, along with long-term follow-up on cerebrospinal fluid and serum markers such as AFP and β-HCG. Additionally, MRI scans should be regularly performed [20]. Because the ostium of dermal sinus tracts is difficult to detect, it is challenging to diagnose it at an early stage. Therefore, these patients should undergo a systematic physical examination, especially on the skin at the central line of the head, back, and lumbosacral region. In addition, MRI scans of the skull and spinal cord are essential. The key to the treatment of the dermal sinus tract is early detection and surgery. The surgery should thoroughly seal the ostium and remove the dural sinus tract to cure the patient. In this case of a mature teratoma, we completely removed it along with the connected arachnoid mater and dermal sinus tract during surgery. The follow-up examination six months after surgery indicated no signs of recurrence. The patient's prognosis was favorable, and the treatment results were satisfactory.

## Conclusions

This study reported a case of an adult with an intracranial mature teratoma comorbid with a dermal sinus tract and subcutaneous lipoma. The disease is clinically rare and has not been reported in the past. Intracranial teratomas are rare and may easily be misdiagnosed before surgery. Because our patient's preoperative images were atypical, he was misdiagnosed as vascular malformation in the cerebellar vermis. After the patient's tumor spontaneously ruptured, the patient's level of consciousness dropped rapidly, and craniotomy was performed. During the surgery, the tumor was observed to be a mixed cystic and solid mass. The liquid in the cyst was a dark-green fatty component. The solid part had a tough texture and comprised hair, fat, cartilage, and calcification components. After surgery, a biopsy proved that the tumor was a mature teratoma. After proactive treatment, the patient's prognosis was favorable.

## References

[1] Lee YH, Park EK, Park YS, Shim KW, Choi JU, Kim DS (2009). Treatment and outcomes of primary intracranial teratoma. Childs Nerv Syst.

[2] Liu Z, Lv X, Wang W (2014). Imaging characteristics of primary intracranial teratoma. Acta Radiol.

[3] Zygourakis CC, Davis JL, Kaur G, Ames CP, Gupta N, Auguste KI, Parsa AT (2015). Management of central nervous system teratoma. J Clin Neurosci.

[4] Goyal N, Kakkar A, Singh PK, Sharma MC, Chandra PS, Mahapatra AK, Sharma BS (2013). Intracranial teratomas in children: a clinicopathological study. Childs Nerv Syst.

[5] Grigore M, Iliev G (2014). Diagnosis of sacrococcygeal teratoma using two and three-dimensional ultrasonography: two cases reported and a literature review. Med Ultrason.

[6] Mete M, Umur AS, Duransoy YK, Barutçuoğlu M, Umur N, Gurgen SG, Selçuki M (2014). Congenital dermal sinus tract of the spine: experience of 16 patients. J Child Neurol.

[7] De Vloo P, Lagae L, Sciot R, Demaerel P, van Loon J, Van Calenbergh F (2013). Spinal dermal sinuses and dermal sinus-like stalks analysis of 14 cases with suggestions for embryologic mechanisms resulting in dermal sinus-like stalks. Eur J Paediatr Neurol.

[8] Wrigh ZG, Rozzelle CJ (2019). Dermal Sinus Tracts.

[9] Singh I, Rohilla S, Kumar P, Sharma S (2015). Spinal dorsal dermal sinus tract: an experience of 21 cases. Surg Neurol Int.

[10] Radmanesh F, Nejat F, El Khashab M (2010). Dermal sinus tract of the spine. Childs Nerv Syst.

[11] Romić D, Raguž M, Marčinković P (2019). Intracranial mature teratoma in an adult patient: a case report. J Neurol Surg Rep.

[12] Zhao J, Wang H, Yu J, Zhong Y, Ge P (2012). Cerebral falx mature teratoma with rare imaging in an adult. Int J Med Sci.

[13] Bhangoo RS, Tammam A, Crockard HA (1997). MRI detection of spontaneous rupture of a well differentiated pineal teratoma. Acta Neurochir (Wien).

[14] Muçaj S, Ugurel MS, Dedushi K, Ramadani N, Jerliu N (2017). Role of MRI in diagnosis of ruptured intracranial dermoid cyst. Acta Inform Med.

[15] Stendel R, Pietila TA, Lehmann K (2002). Ruptured intracranial dermoid cysts. Surg Neurol.

[16] Lunardi P, Missori P (1991). Supratentorial dermoid cysts. J Neurosurg.

[17] Shashidhar A, Sadashiva N, Prabhuraj AR (2019). Ruptured intracranial dermoid cysts: a retrospective institutional review. J Clin Neurosci.

[18] Liu JK, Gottfried ON, Salzman KL, Schmidt RH, Couldwell WT (2008). Ruptured intracranial dermoid cysts: clinical, radiographic, and surgical features. Neurosurgery.

[19] Hashiguchi K, Inamura T, Nishio S, Nakamizo A, Inoha S, Fukui M (2001). Mobile intracranial oily substances from a ruptured teratoma. J Clin Neurosci.

[20] Murray MJ, Bartels U, Nishikawa R (2015). Consensus on the management of intracranial germ-cell tumours. Lancet Oncl.

